# A Clinical Comparison of Failure Rates of Metallic and Ceramic Brackets: A Twelve-Month Study

**DOI:** 10.1155/2020/9725101

**Published:** 2020-01-10

**Authors:** Tomasz Ogiński, Beata Kawala, Marcin Mikulewicz, Joanna Antoszewska-Smith

**Affiliations:** ^1^Department of Maxillofacial Orthopaedics and Orthodontics, Division of Facial Abnormalities, Wrocław Medical University, Wrocław 50-425, Poland; ^2^Department of Maxillofacial Orthopaedics and Orthodontics, Wrocław Medical University, Wrocław 50-425, Poland

## Abstract

**Objective:**

Clinical comparison of the survival rates between stainless steel and ceramic brackets over a 12-month period.

**Materials and Methods:**

The study involved 20 consecutive patients with diagnosed malocclusion that required two-arch fixed appliance treatment. The participants were randomly divided into two 10-member groups. Group 1 was treated with Abzil Agile (3M Unitek) stainless steel brackets; group 2 was treated with Radiance (American Orthodontics) monocrystalline ceramic brackets. All the brackets were bonded by the same operator. Over the next 12 months, all bracket failures were recorded with each appointment. The received data were processed statistically using the Mantel–Cox test, Kaplan–Meier method, and Cox hazard model.

**Results:**

A total of 381 brackets were bonded, 195 of which were metallic brackets and 186 were ceramic ones. In the 12-month observation period, there were 14 metal (7.2%) and 2 ceramic bracket (1.1%) failures. The overall failure rate was 4.2% (*n* = 16). The majority of failures (14 brackets; 87.5%) occurred during the first 6 months of the experiment, 12 (83%) of which were metal brackets and 2 (100%) were ceramic brackets. The statistical analysis revealed significant differences between the groups (*p* < 0.05).

**Conclusions:**

Metal brackets demonstrated significantly higher failure rates than ceramic brackets for both 6- and 12-month observation periods (*p* < 0.05). The 6% difference between the brackets is clinically significant as it corresponds to one additional failure within 12 months.

## 1. Introduction

Orthodontic bracket is an essential element of fixed appliance. Its purpose is to transfer forces from the activated archwire to dentition to enable three-dimensional movement of teeth. Currently, stainless steel brackets are most commonly used at the orthodontic office due to their low cost, high corrosion resistance in the mouth, higher modulus of elasticity, and excellent biomechanical properties [1, 2]. Since stainless steel cannot bond chemically with orthodontic adhesives, these brackets have different types of gauge mesh bases for increasing the contact area with the adhesive. During bracket positioning, mesh eyelets are filled with orthodontic adhesive, and the subsequent polymerisation creates a micromechanical bond between the bracket and the adhesive [3]. In addition to numerous advantages, stainless steel brackets also have some drawbacks, which are poor aesthetics and low biocompatibility. Both clinicians and patients are aware of this problem, which leads to increased interest in ceramic brackets due to their cosmetic properties and high biocompatibility [2]. However, ceramic materials, just like stainless steel, do not form chemical compounds with acrylic and diacrylate orthodontic adhesives [4]. Bases of ceramic brackets are usually formed with recesses or covered with additional ceramic particles to ensure a better mechanical interlock to the adhesive. Another method is to coat the ceramic base with silane to provide chemical adhesion [5, 6].

Bond strength of orthodontic brackets is an important factor which can influence the treatment with the use of fixed appliances. Bracket failures may potentially increase the total treatment time and financial costs of the therapy. The optimal bonding force between the bracket and enamel surface should be sufficient to enable a durable bracket position during treatment and to prevent the enamel from iatrogenic damage during the debonding procedure. Bond failures may be caused by numerous factors, including masticatory forces, forces produced by orthodontic appliances, aging of the orthodontic adhesives, mistakes during any step of bonding protocol, or some conservative dentistry therapies performed prior to bonding, such as topical fluoride varnish applications, or bleaching [7–9]. The range of the desired bonding force has not been determined yet. On the basis of their *in vitro* study, Reynolds and von Fraunhofer [10] stated that the minimum bond strength of 6–8 MPa is considered appropriate, whereas Bishara [11] suggested that the bonding strength ought to exceed 13.5 MPa. Recently Gauge [3] assumed that the ideal orthodontic adhesive should withstand forces over 20 MPa. The majority of the studies that evaluated the bond strength of orthodontic brackets were carried out as *in vitro* experiments under ideal laboratory conditions that may not reflect all clinical conditions. *In vitro* experiments provide information about initial bond strength to the enamel but cannot serve as predictors of bracket survivability [12–14]. Therefore, more accurate guidance on the clinical relevance of adhesion protocols is provided by *in vivo* tests, which assess the failure rate of the enamel-boding agent-bracket interface during treatment.

Bracket failure occurs at one of the three locations within the enamel-adhesive-bracket complex: between the tooth enamel and the adhesive, within the adhesive, or at the adhesive-bracket interface. The adhesive-enamel interface has been well tested as evidenced by numerous articles dedicated to both *in vivo* and *in vitro* tests. These surveys mostly assessed the application of self-etching primer which is still controversial [15–19]. The evaluation of identical adhesive systems and two different orthodontic brackets makes it possible to compare the bond strength between the adhesive and the bracket. However, there are few inconsistent results of *in vitro* tests that compare the bond strength of metal and ceramic brackets with the enamel. Some authors do not observe statistically significant differences between metal and ceramic brackets subjected to shear or tension bond strength testing [20, 21]. On the other hand, Joseph and Russouw [22], as well as Haydar et al. [23], obtained significantly higher mean values of the shear bond strength of ceramic brackets in comparison to their metal counterparts for all tested bonding materials. Regardless of the results of statistical tests, the bond strength of metal and ceramic brackets measured by all the authors exceeded 6–8 MPa, which is considered to be adequate for clinical use [23].

The purpose of this *in vivo* study was to investigate and compare failure rates of ceramic and stainless steel brackets over a period of 12 months.

## 2. Materials and Methods

**Figure 1:**
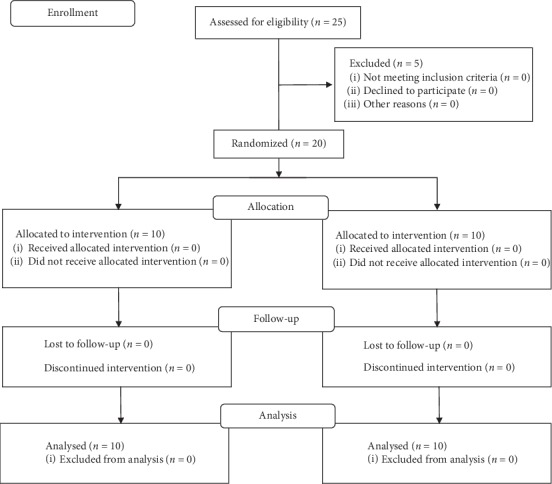


The study involved 20 consecutive patients with diagnosed malocclusion that required two-arch fixed appliance treatment (Figure 1). Completely erupted anterior teeth and premolars were the only inclusion criteria for this study. Patients with enamel defects, fillings, or prosthetic work on buccal surfaces of teeth were excluded from the study. The subjects treated with adjunctive fixed functional appliances (Herbst, Forsus, etc.) were also excluded from the study due to an increased failure rate of lower canine brackets located near their mandibular attachments, which was observed in our practice. Age, gender, and malocclusion differences were ignored. Both extraction and nonextraction patients participated in the study. 16 out of 20 patients were subjected to treatment without extraction; 4 patients had either first or second upper premolars extracted. Furthermore, two patients had the pair of upper premolars extracted in childhood (interview data), and 4 patients were diagnosed with hypodontia of 7 teeth in total. Thus, the study was conducted in relation to a total of 381 teeth. The study was approved by the ethical committee of Wrocław Medical University (181/2013).

The participants were randomly divided into two groups that consisted of 10 subjects in each group. Group 1 was treated with Agile (3M Abzil, São José do Rio Preto, Brazil) stainless steel brackets; group 2 was treated with Radiance (American Orthodontics, Sheboygan, USA) monocrystalline ceramic brackets. No patient was lost to follow-up. The average age of the patients was 25.8 years (range: 13–44 years) (Table 1). Due to the nature of the experiment, both the operator and the patient knew whether they were treated with metal or ceramic brackets.

All the brackets were bonded by the same operator (TO) in the following manner. After a lip and cheek retractor had been placed, dental biofilm was removed with a brush mounted on a micromotor without using prophylaxis or abrasive paste. Then, Transbond Plus Self Etching Primer (3M Unitek, Monrovia, USA) was rubbed into the clean enamel for 5 seconds. The excessive amount of the solvent was vaporized by an air stream from an oil-free air-water syringe. Afterwards, a thin layer of Transbond XT (3M Unitek, Monrovia, USA) orthodontic adhesive was put on the bracket base. A bracket was positioned on the enamel surface, whereas the excessive amount of the adhesive was removed with a probe. As recommended by the Transbond XT manufacturer, the adhesive was being cured for 20 seconds with a diode polymerisation lamp and 10 seconds for the mesial and distal sides each in the case of applying a metallic bracket or through a bracket in the case of applying a ceramic bracket. The initial archwire was inserted immediately after bracket bonding. If the contact between the upper teeth and the mandibular bracket was observed, an elastomeric ligature with guard (3M Unitek, Monrovia, USA) was applied to the lower bracket, as, according to our clinical experience, it brings sufficient protection from the failures. During the bonding appointment, the patients were instructed how to take care of the fixed appliance and how to maintain proper hygiene of the oral cavity during treatment.

All brackets bonded to the incisors, canines, and premolars were evaluated. During the first 12 months of active treatment, at each appointment, the orthodontist (TO) recorded all the bracket failures. If a failure occurred, the orthodontist bonded a new bracket that was not included in the study. The obtained data were subjected to statistical analysis using the Mantel–Cox test. The correlation between treatment time and failure rate was evaluated with the Kaplan–Meier method. Risk factors were computed using the Cox proportional hazards model. The level of statistical significance for all tests was set at *α* = 0.05.

## 3. Results

A total of 381 brackets were bonded, 195 of which were metallic brackets and 186 were ceramic ones. In the 12-month observation period, there were 14 metal (7.2%) and 2 ceramic bracket (1.1%) failures. The overall failure rate was 4. 2% (*n* = 16). The majority of failures (14 brackets; 87.5%) occurred during the first 6 months of the experiment, 12 (83%) of which were metal brackets and 2 (100%) were ceramic brackets (Table 2). Bracket failure rates were 6.25% for canines, followed by 5% for central incisors. The lowest number of detachments was encountered at the second premolar with failure rate of 1.3% (Table 3).

**Figure 2:**
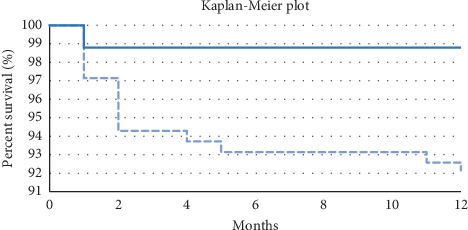
Kaplan–Meier survival plots. Continuous line: ceramic bracket; dashed line: stainless steel bracket.

The log-rank test showed significant statistical differences between the brackets for both 6- and 12-month observation periods, *p*=0.008 and *p*=0.003, respectively. The Kaplan–Meier survival curve plotted for the 12-month observation period showed a greater tendency towards metal bracket failure occurring from the beginning of the treatment (Figure 2). The Cox proportional hazards model showed for both 6- and 12-month observation periods that metal brackets have a 15% greater risk of failure than ceramic ones (*p* < 0.05) (Table 4).

## 4. Discussion

This study investigated the detachment rates of the ceramic and metallic brackets. The failure rate of the ceramic brackets in our study was 1.1%. To date, no data have been reported in relation to the failure rates of the ceramic brackets bonded with composite adhesive and self-etching primer. Hitmi et al. [24] reported a similar failure rate of 0.7% for the ceramic brackets bonded with Fuji Ortho LC, light-cured glass-ionomer resin-modified adhesive. Higher values were obtained by Årtun [25], who compared the failure rates of the ceramic brackets with mechanical or chemical retention and found 1.7% and 3.2%, respectively. Recently, Stasinopoulos et al. [26], in retrospective study, reported a detachment rate of the ceramic brackets of 20%. The authors, however, included in the survey only the patient with at least one bracket failure during the orthodontic treatment.

The failure rate of the metal brackets obtained in our study was 6.2%, and it is within the range of the results (1.15%–17.87%) found by other authors [9, 14, 16, 24, 27–33].

A direct comparison of the study results is difficult due to the different experiment protocols concerning brackets, adhesive systems, tooth enamel preparation, curing time, curing power, number of operators, length of the study, and the age of the patients. In this study, the failure rate of the metal brackets was seven-times higher than that of the ceramic brackets and this difference was statistically significant (*p* < 0.05). These results are in agreement with the study by Hitmi et al. [24], who compared the detachment rates of the metal, plastic, and ceramic brackets bonded with resin-modified glass-ionomer adhesive and, similarly to our study, discovered a statistically larger percentage of failures of metal brackets than ceramic ones. The difference in the failure rates between the metallic and ceramic brackets found in our study is hard to explain. In the laboratory study, Benkli et al. [34] showed that shear bond strength of the Radiance bracket bonded to human enamel is twice as high as that of the metallic ones. On the other hand, Oginski at al. [35] did not find statistical differences between the bond strength of Radiance brackets and metallic brackets bonded with the self-priming system and colour changing Grengloo adhesive to bovine enamel. Despite the conflicting results of the *in vitro* investigations, the difference in the failure rates may be contributed to the different degree of cure of the orthodontic adhesive under brackets made from different materials. Eliades et al. [36] investigated the degree of cure of the adhesive under brackets under the irradiation modes used in our study. They found a significantly higher degree of cure of directly irradiated orthodontic light-cured adhesive under monocrystalline brackets in comparison with the indirectly irradiated adhesive under stainless steel attachments. Moreover, monocrystalline brackets had a diffuse visible light transmittance of 80% at 468 nm compared to almost no light transmittance of metallic brackets. Therefore, during the bonding procedure, curing of orthodontic adhesive under ceramic brackets will lead to additional polymerisation of adhesive under previously bonded brackets, resulting in an even more initial difference between the degrees of cure of adhesives under ceramic and stainless steel brackets.

In our investigation, we found that the detachment rate of the second premolar bracket was 1.4% and was lower in comparison with the failure rates of the anterior teeth. These results are in contradiction with numerous researchers that reported higher failure rates in the posterior region [9, 14, 37–39]. This is attributed to higher masticatory forces in posterior region, problems with moisture control, poor access, bracket-tooth contact after bonding, and increased amount of prismatic enamel [37]. On the other hand, Krishnan et al. [33] noted 1.7 times higher failure rate in the anterior region. Recent survey focused on the evaluation of patterns of bracket failure relates this to the increased activation forces exerted by an archwire in the crowded dental arches or increased mastication load [26].

The failure rate of the metal brackets is reported to be much higher during the first 6 months of the treatment [16, 38, 40, 41]. In the studies where the failure rates of the metal brackets bonded with the self-etching primer system were analysed after 6 and 12 months of treatment, it was found that 50–68% of detachments occurred within the first half year of the therapy [15, 42]. In this study, we obtained a higher failure rate of 83%. The difference may be due to the fact that, contrary to other authors [15, 42], we did not apply the pumice paste to the enamel prior to bonding brackets: enamel biofilm was only removed with a brush mounted on a micromotor without using prophylaxis or abrasive paste. We have not found studies comparing the applied procedure with other methods, i.e., cleaning tooth enamel with pumice or refraining from cleaning it completely, but Ireland and Sheriff [43] found in an *in vivo* study that the application of pumice prior to enamel etching does not improve the bond strength of the metal brackets. Similar results obtained from *in vitro* experiments were reported by others [44], also in the case of applying a self-etching primer [45]. On the other hand, Lill et al. [46] observed a five-time increase in the bracket failures in the patients whose enamel had not been cleaned with pumice prior to the application of self-etching primer.

A limitation of our study is that we did not evaluate the failure rate of dental brackets by gender, age, or malocclusion. The evidence in relation to the patient's gender and bracket survival is ambiguous. Numerous researchers reported no difference in the bracket failure rate between males and females [15, 24, 27, 31, 37, 41, 42, 47, 48]. On the other hand, Koupis et al. [49], Hammad et al. [41], and Jung [29] reported a significantly higher failure rate in males than females, whereas Bazagrani et al. [32], who examined the bracket failure rate among adolescents, found that detachments of brackets in boys occurred four times more frequently than in girls.

Also, the relation between the bracket failure rates and the patient's age is disputable. Jung [29] discovered that adolescents have a higher rate of bracket failures compared to adults. Recently, Bazagrani et al. [32] reported a 3.5 times higher detachment rate among adolescents aged 10–13 than those at the age of 14–18. Other authors did not find a statistical difference for the failure rate with respect to the age of the patient [40, 45, 50].

Likewise, the effect of the malocclusion on the bracket detachments rate is questionable. Although Bherwani et al. [27] reported that class II/2 malocclusion significantly increases the likelihood of bracket failure, other authors did not find significant differences [41, 51]. In our study, we applied an elastomeric ligature with guard (3M Unitek) to every bracket that was in occlusal contact with the opposing teeth after bonding, which might lead to the bond failure.

Another limitation of this study is related to the fact that all brackets were bonded by the same orthodontist (TO). Thus, according to Nandhra at al. [31], the results of this investigation may only be attributed to that clinician. Also, due to a clearly visible difference in colour between stainless steel and ceramic brackets, neither the operator nor the patient could have been blinded in terms of the type of brackets used during the treatment.

Bearing in mind the diversity of ceramic bracket bases, extreme caution should be exercised in generalizing our findings and extrapolating them to other ceramic brackets, especially when differences between the construction of ceramic bases are a known factor that has a significant impact on the results of *in vitro* bond strength studies [52].

In the modern era, with the technology development and the associated diversity of both orthodontic attachments and bonding materials, studies must be carried out to evaluate the usefulness of constantly improved orthodontic products. Since *in vitro* tests cannot exactly reflect clinical conditions, further *in vivo* studies are necessary to develop clinically acceptable adhesion protocols for orthodontic brackets.

## 5. Conclusions

This clinical study showed that metal brackets exhibited significantly higher failure rates than ceramic brackets for both 6- and 12-month observation periods (*p* < 0.05). The 6% difference between the failure rates of tested brackets is clinically significant as it corresponds to one additional failure within 12 months. The Cox proportional hazard model showed that metal brackets have a 15% greater risk of failure than ceramic ones (*p* < 0.05). However, both ceramic and metal brackets bonded with self-etching primer had acceptable failure rates, and both of them can be recommended to clinicians.

The majority of bracket failures (87.5%) occurred during the first six months of the treatment.

## Figures and Tables

**Table 1:** Gender and age distribution between groups.

	Males, *n*, %	Females, *n*, %	Malocclusion			Age	
			Class I, *n*, %	Class II, *n*, %	Class III, *n*, %	Mean, ys	Range, ys
Metallic bracket	2 (20)	8 (80)	5 (50)	5 (50)	0 (0)	21.7	13–33
Ceramic bracket	2 (20)	8 (80)	4 (40)	6 (60)	0 (0)	30.1	16–44
Total	4 (20)	16 (80)	9 (45)	11 (55)	0 (0)	25.7	13–44

**Table 2:** Number and percentage of failed brackets.

Bracket type	Bonded, *N*	Failures after 6 months, *N*	Failures after 12 months, *N*	Failures after 12 months, %
Metallic	195	12	14	7.2
Ceramic	186	2	2	1.1
Total	381	14	16	4.2

**Table 3:** Bracket failure rates per tooth type after 12-month observation period.

Tooth type	Brackets bonded	Failures after 12 months	Failure rate (%)
Mesial incisor	80	4	5
Lateral incisor	79	3	3.8
Canine	80	5	6.25
First premolar	68	3	4
Second premolar	74	1	1.3

**Table 4:** The Cox proportional hazards model for 6- and 12-month observation periods.

	Coefficient *β*	Hazard ratio	95% CL	*p*
Ceramic brackets (6 months)		1.00	1.00	
Metal brackets (6 months)	−1.752	0.173	0.039–0.775	0.022
Ceramic brackets (12 months)		1.00	1.00	
Metal brackets (12 months)	−1.913	0.148	0.034–0.650	0.011

## Data Availability

The data used to support the findings of this study are included within the article.
